# The “psychiatric” neuron: the psychic neuron of the cerebral cortex, revisited

**DOI:** 10.3389/fnhum.2024.1356674

**Published:** 2024-03-18

**Authors:** L. Taylor Flynn, Nadia N. Bouras, Volodar M. Migovich, Jacob D. Clarin, Wen-Jun Gao

**Affiliations:** ^1^Department of Neurobiology, Drexel University College of Medicine, Philadelphia, PA, United States; ^2^Drexel University College of Medicine, Philadelphia, PA, United States

**Keywords:** prefrontal cortex, Psychic cells, working memory, social behavior, schizophrenia, ADHD, anxiety, mental disorders

## Abstract

Nearly 25 years ago, Dr. Patricia Goldman-Rakic published her review paper, “The ‘Psychic’ Neuron of the Cerebral Cortex,” outlining the circuit-level dynamics, neurotransmitter systems, and behavioral correlates of pyramidal neurons in the cerebral cortex, particularly as they relate to working memory. In the decades since the release of this paper, the existing literature and our understanding of the pyramidal neuron have increased tremendously, and research is still underway to better characterize the role of the pyramidal neuron in both healthy and psychiatric disease states. In this review, we revisit Dr. Goldman-Rakic’s characterization of the pyramidal neuron, focusing on the pyramidal neurons of the prefrontal cortex (PFC) and their role in working memory. Specifically, we examine the role of PFC pyramidal neurons in the intersection of working memory and social function and describe how deficits in working memory may actually underlie the pathophysiology of social dysfunction in psychiatric disease states. We briefly describe the cortico-cortical and corticothalamic connections between the PFC and non-PFC brain regions, as well the microcircuit dynamics of the pyramidal neuron and interneurons, and the role of both these macro- and microcircuits in the maintenance of the excitatory/inhibitory balance of the cerebral cortex for working memory function. Finally, we discuss the consequences to working memory when pyramidal neurons and their circuits are dysfunctional, emphasizing the resulting social deficits in psychiatric disease states with known working memory dysfunction.

## Introduction

Patricia Goldman-Rakic was an accomplished neuroscientist and dedicated researcher whose innovative and multidisciplinary investigative approach permitted extensive characterization of the prefrontal cortex (PFC), despite the belief of the time that such a brain region was largely impossible to probe experimentally. Dr. Goldman-Rakic was relentless in her pursuits to understand the role of the PFC in cognition, and particularly in functions of working memory. Working memory, or the ability to transiently hold and manipulate information, is a vital component of cognitive function that underlies innumerable facets of behavior across species (D’Esposito and Postle, 2015). Working memory can be subdivided based on the sensory modalities of the presented stimuli, including visuospatial, auditory, and verbal working memory, and the particulars of the neural pathways involved will vary based on the assessment utilized. Additionally, working memory can be conceptualized under different theoretical umbrellas, and recent frameworks posited to explain working memory include processes of synaptic facilitation, astrocytic regulation, and intrinsic network dynamics (Durstewitz et al., 2000; Mongillo et al., 2008; Barak and Tsodyks, 2014; Gordleeva et al., 2021). Regardless of the specific facet of working memory being tested, however, adequate function and connectivity of the PFC is necessary for task performance (Goldman-Rakic and Friedman, 1991; Goldman-Rakic, 1995).

Dr. Goldman-Rakic’s research elegantly demonstrated the invaluable role of the PFC in working memory function, supported by its integrative role in the higher order processing of sensory information, and highlighted the unique qualities of PFC neurons that make them so well-suited for this role (Goldman-Rakic, 1995). Chief among these qualities was the ability of the pyramidal neuron, the principal neuron of the PFC, to generate and maintain persistent activity beyond the period of stimulus exposure (Funahashi et al., 1991; Riley and Constantinidis, 2015). In the oculomotor task of working memory, individual PFC neurons were shown to respond to a visual stimulus within a narrow section of the visual field, with nearby neurons thought to respond to similar visual field sections, ultimately forming a cortical column that acts cooperatively to process a particular stimulus (Funahashi et al., 1989). These PFC pyramidal neurons demonstrate persistent activity that is maintained for a short period of time (up to several dozen seconds) following presentation of the stimulus, and this persistent activity has been heavily implicated in working memory function (Wang, 1999; Wang et al., 2007, 2013; Constantinidis et al., 2018; Kilonzo et al., 2021).

Modeling work by Xiao-Jing Wang in 1999 first suggested a role for slow transmission mediated by N-methyl D-aspartate (NMDA) receptors in the maintenance of PFC PC persistent activity (Wang, 1999). This was later supported by experimental work in Amy Arnsten’s laboratory, which showed that local blockade of NMDARs in the primate PFC resulted in a reduction in persistent activity (Wang et al., 2013). Furthermore, the group showed that systemic blockade of NMDARs by ketamine administration reduced working memory performance in these animals (Wang et al., 2013). However, it was not until 2021 that the gap between molecular and behavioral effects of NMDAR inhibition was bridged, when work by Kilonzo and colleagues demonstrated that NMDAR knockdown in PFC PCs specifically resulted in decreased working memory performance (Kilonzo et al., 2021). While there remains some debate in the field, a recent review by Amy Arnsten’s group argued that, in the face of such overwhelming evidence for the role of PFC PC persistent activity in working memory function, individual negative studies should be viewed cautiously (Constantinidis et al., 2018). Early on, Dr. Goldman-Rakic recognized the significance of this capacity for persistent activity, and much of her work centered on elucidating the intricacies of PFC PCs and their role in working memory (Goldman-Rakic, 1999).

More recent research has focused on the dysfunctions that arise as a result of perturbations of this brain region, including the role of working memory deficits in a number of psychiatric conditions. Here, we aim to provide an update to Dr. Goldman-Rakic’s characterization of the PFC pyramidal neuron and its role in working memory, as well as specifically examine the role of working memory dysfunction in the pathophysiology of social deficits. First, we will describe the macro- and microcircuitry of the PFC and the role of this circuitry in the maintenance of excitation/inhibition balance amongst PFC pyramidal neurons in working memory function. We will then highlight the importance of working memory in social functioning by examining the perturbations of this circuitry in disorders of the PFC and the resulting social deficits observed.

## PFC macrocircuits and working memory

**Figure 1:**
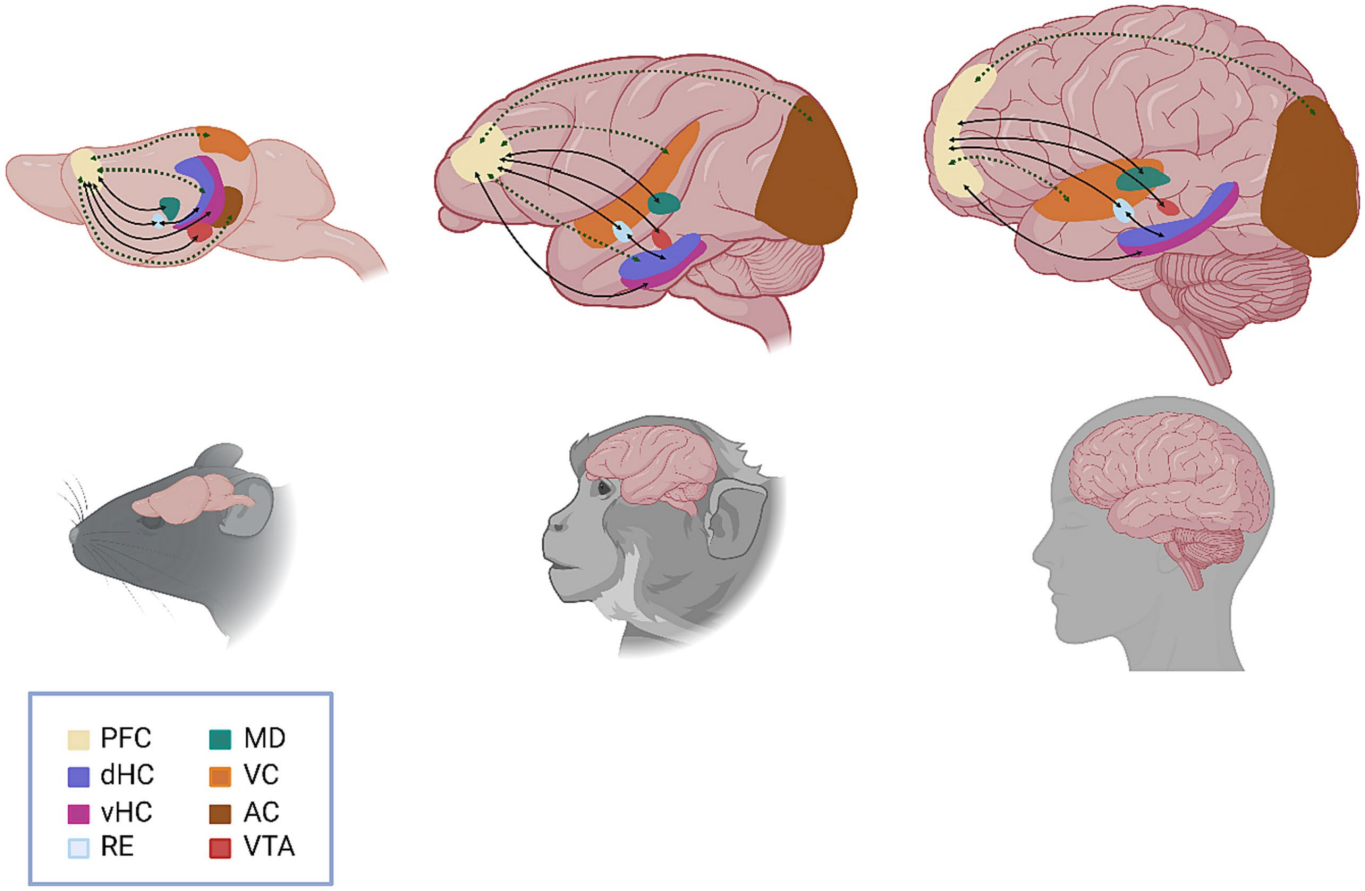
Schematic representation of the functional circuits involved in working memory. Anatomical (solid black line) and functional (dashed green line) connections between brain regions are represented by arrows, with arrowheads indicating the direction of the connection. Specific circuits highlighted include VTA to PFC (direct, reciprocal); vHC to PFC (direct) and dHC to PFC (indirect/multisynaptic); PFC to RE (direct, reciprocal) and RE to dHC (direct, reciprocal); PFC to MD (direct, reciprocal); VC to PFC (indirect/multisynaptic); and AC to PFC (indirect/multisynaptic). PFC, prefrontal cortex; dHC, dorsal hippocampus; vHC, ventral hippocampus; RE, nucleus reuniens, MD, mediodorsal thalamus; VC, visual cortex; AC, auditory cortex; VTA, ventral tegmental area.

Amongst the most significant scientific contributions by Patricia Goldman-Rakic during her long career was the wealth of anatomical studies she conducted in nonhuman primates to elucidate the connections between the PFC and other cortical and subcortical brain regions and the role of these pathways in working memory (Arnsten, 2023). Indeed, from her seminal 1970 paper identifying distinct subdivisions of the dorsolateral PFC, which implicated specifically the principal sulcus of the dorsolateral PFC in working memory function (Goldman and Rosvold, 1970), Dr. Goldman-Rakic’s work served to lay the groundwork for the identification and characterization of the functional connectome of the PFC with task- and function-specific clarity. Recent technical strides within the field of neuroscience have allowed for the dissection of the circuits involved in working memory with fine spatiotemporal resolution. These studies advocate for a framework wherein no particular brain region can be deemed the sole “locus” of working memory, but instead demonstrate how the PFC functions within broader neural networks to support this phenomenon. Here, we review recent advances in working memory research from a connectome perspective to highlight the function of the prefrontal pyramidal neuron and the broader networks in which it operates (Figure 1).

While Dr. Goldman-Rakic’s early research demonstrated the critical role of the PFC in working memory function, a growing body of evidence additionally supports the role of other cortical regions, including sensory, temporal, and parietal cortices, and suggests that the recruitment of specific cortical regions is often task-dependent (Wager and Smith, 2003; Pasternak and Greenlee, 2005). One contemporary model integrating these observations asserts that working memory is subserved by a distributed cortical network that encodes multiple levels of abstraction of a particular stimulus (Christophel et al., 2017). Considering complex working memory tasks from the perspective of this model, sensory cortices must maintain low-level sensory information. In contrast, prefrontal and associative cortices are instead tasked with the representation of categorical and semantic contents of a stimulus. The question then becomes, how do cortical regions communicate to effectively integrate multiple levels of stimulus encoding to orchestrate appropriate behavioral responses for working memory? Several studies already suggest that communication between the PFC and other cortical regions is a feature of certain working memory tasks (Liebe et al., 2012; Salazar et al., 2012; Jacob and Nieder, 2014; Murray et al., 2017), however, the precise integrative mechanisms of this communication are not yet understood.

In addition to cortico-cortical interactions, the influence of subcortical communication with the PFC on working memory function and on adaptive behavior more generally cannot be overstated. Indeed, to support adequate working memory function, the PFC is fine-tuned by neuromodulators, such as dopamine, that originate primarily from subcortical structures. The ventral tegmental area (VTA), a midbrain structure containing PFC-projecting dopaminergic cell bodies, is a primary source of dopamine in the PFC and is implicated in a range of cognitive and emotional processes (Seamans and Yang, 2004). While Dr. Goldman-Rakic’s lab pioneered the seminal studies that supported the importance of dopaminergic signaling in the PFC for working memory function (Sawaguchi and Goldman-Rakic, 1994), subsequent research capitalizing on the genetic tractability of mouse models has revealed more intricate details regarding VTA-PFC communication (Duvarci et al., 2018). For example, Ge and colleagues utilized temporally precise optogenetic manipulation to discern that dopaminergic modulation of the PFC at early and late-delay period epochs results in differential effects on working memory performance (Ge et al., 2023).

In 1984, following a series of anterograde and retrograde tracing studies demonstrating the reciprocal pathways connecting the dorsolateral PFC to the hippocampus and parahippocampal gyrus in rhesus monkeys, Goldman-Rakic and colleagues developed a new hypothesis. They theorized that, in addition to direct projections from the hippocampus to the PFC, there exist indirect (polysynaptic) connections from the PFC to the hippocampus and associated cortices that can be divided into a medial and lateral pathway, each of which carries distinct information (Goldman-Rakic et al., 1984). In the years following that initial hypothesis, extensive research into PFC-hippocampal communication elucidated the critical role of these two brain regions in working memory function. Rodent anatomical and electrophysiological studies have shown divergent cortical connectivity and function between the dorsal (dHC) and ventral hippocampus (vHC) (Bannerman et al., 2004; Fanselow and Dong, 2010; Strange et al., 2014). Hippocampal-medial (m)PFC afferent communication occurs via unidirectional, monosynaptic projections from the CA1/subiculum of the vHC and via strictly indirect, multi-synaptic connections from the dHC (Laroche et al., 2000; Thierry et al., 2000; Cenquizca and Swanson, 2007; Hoover and Vertes, 2012). In terms of working memory, increased theta- and gamma-frequency synchrony is observed between both the vHC-PFC and dHC-mPFC circuits during spatial working memory tasks (Jones and Wilson, 2005; Hyman et al., 2010; Sigurdsson et al., 2010; Spellman et al., 2015; Salimi et al., 2022). Specifically, data suggest that gamma-oscillation synchrony between vHC-PFC neurons is critical for accurate spatial working memory encoding (Spellman et al., 2015), while increased delta, but not gamma, synchrony was observed during correct trials in a spatial working memory task (Salimi et al., 2022). Additionally, engagement in working memory tasks has been shown to modulate theta-synchrony in both vHC-PFC and dHC-PFC circuits (O’Neill et al., 2013). Furthermore, inactivation of the vHC leads to reduced dHC-PFC theta-oscillation synchrony, suggesting that vHC activity modulates dHC-PFC synchrony during working memory (O’Neill et al., 2013). While there is no evidence to date that oscillatory coherence is sufficient to guide working memory, these results nonetheless advocate for a dynamic network model in which PFC-hippocampal communication is modulated when animals are placed in behavioral contexts evoking working memory. Furthermore, it should be noted that alterations in oscillatory activity have been associated with working memory deficits in psychiatric disease, such as the co-occurrence of aberrant gamma synchrony and notable working memory deficits in schizophrenia (Dienel and Lewis, 2019).

Growing anatomical evidence suggests that one of the key mediators of the polysynaptic connection between the PFC and hippocampus is the thalamic nucleus reuniens (Re), a brain region that is reciprocally connected to the mPFC and hippocampus (Wouterlood et al., 1990; Vertes, 2002, 2006). Notably, a small subset of Re neurons that receive mPFC input send direct projections to the hippocampus (Vertes, 2006; Vertes et al., 2006; Varela et al., 2014). Moreover, 25% of hippocampus-projecting Re neurons also project to the mPFC (Varela et al., 2014). Inactivation of the Re disrupts oscillatory synchrony in CA1-mPFC projections (Hallock et al., 2016) and results in working memory deficits (Hembrook and Mair, 2011; Hembrook et al., 2012; Cholvin et al., 2013; Hallock et al., 2013; Duan et al., 2015; Layfield et al., 2015; Hallock et al., 2016; Maisson et al., 2018; Viena et al., 2018). Thus, it is posited that the hippocampal-PFC synchrony observed during working memory may be mediated by the Re (Vertes et al., 2006; Griffin, 2015; Dolleman-van der Weel et al., 2019).

As demonstrated by Goldman-Rakic’s laboratory in 1982, reciprocal connectivity also exists between the mPFC and the mediodorsal thalamus (MD) (Kievit and Kuypers, 1977; Isseroff et al., 1982; Goldman-Rakic and Porrino, 1985; Giguere and Goldman-Rakic, 1988). Specifically, augmented beta oscillation synchrony between the MD and mPFC has been observed during both the acquisition and performance of working memory tasks. Further, increases in beta synchrony between these two regions correspond to improvements in working memory performance (Parnaudeau et al., 2013, 2018). Inhibition of either MD-mPFC alone in rats (Ferguson and Gao, 2018) or both MD-mPFC and mPFC-MD pathways in mice results in decreased working memory performance. One working model is that elevated, activity in the mPFC during the delay period in working memory tasks is supported by inputs from MD, and that top-down communication from the mPFC to the MD is important for memory retrieval and action selection (Bolkan et al., 2017). This model is supported by both rodent and primate studies, given that silencing of dorsolateral PFC-lateral MD projections impaired performance in a spatial working memory task, and that, conversely, enhancing MD activity optogenetically during the delay period results in improved task performance (Bolkan et al., 2017; Oyama et al., 2021). Together, these circuit studies show that the PFC does not act alone to support working memory, but instead works in concert with other brain regions to learn, maintain, and express working memory task-related information.

## PFC microcircuitry and working memory

Beyond characterizing the long-range afferent inputs and efferent projections from the PFC that comprise the working memory macrocircuit, Goldman-Rakic was also interested in describing the unique neuronal morphological characteristics and microcircuitry within the PFC that permitted its unique ability for persistent activity. Pyramidal neurons within layer III of the PFC have particularly dense apical arborizations, which permit spatial summation of incoming excitatory potentials from both neighboring pyramidal neurons (Kritzer and Goldman-Rakic, 1995; Wang et al., 2011) and distant brain structures involved in working memory, such as the hippocampus (Abbas et al., 2018). Additionally, these layer III pyramidal neurons express relatively high levels of NR2B-containing N-methyl D-aspartate (NMDA) receptors, which have long activation kinetics and allow for slower decay of incoming activation (Wang, 1999; Wang et al., 2008, 2013). In early development, these NMDA subunits are present in many cortical areas and neuronal cell types, where they contribute to early-life plasticity (Monaco et al., 2015). In the rodent PFC, these subunits are present on pyramidal neurons and are thought to contribute to functions of working memory and decision-making (Murphy et al., 2005; Dalton et al., 2011). In macaque, pharmaceutical blockade of these subunits, but not of NR2A subunits, results in ablation of persistent firing during the delay period (Wang et al., 2013). Furthermore, dysregulation of NMDAR function generally and of NR2B expression specifically have been implicated in the pathophysiology of schizophrenia, and may underlie some of the working memory deficits observed in this disorder (Kantrowitz and Javitt, 2010).

In addition to the modulation of excitatory transmission inherent in the persistence of the NR2B subunit, PFC pyramidal neurons are part of a complex microcircuitry that works to maintain the excitation level of the cortical column as it is engaged in tasks of working memory. Goldman-Rakic identified not only the microcircuit between individual excitatory pyramidal neurons of the PFC, demonstrating that the mediolateral columnar organization of neurons within and across cortical layers differed from that of other cortical regions, such as the primary visual cortex (Kritzer and Goldman-Rakic, 1995), but additionally recognized the importance of inhibition within this microcircuit. Indeed, while the large excitatory pyramidal neurons of the PFC have been the focus of early work in working memory, a small percentage (~15–17%) of PFC neurons release inhibitory gamma-aminobutyric acid (GABA) (Rudy et al., 2011). These inhibitory interneurons (INs) are smaller and canonically projected locally; thus, their arrangement defines the local PFC microcircuit. To this end, Constantinidis et al. (2002) used simultaneous recordings in monkeys and reported an important role of inhibition in the cerebral cortex-controlling the timing of neuronal activities during cognitive operations and thereby shaping the temporal flow of information in the PFC. The PFC microcircuit was further modeled by Dr. Goldman-Rakic and her laboratory as consisting of four cell types: pyramidal neurons, perisoma-targeting PV neurons, dendrite-targeting calbindin (CB) neurons, and interneuron-targeting calretinin (CR) neurons (Wang et al., 2004). They predicted that CB INs would demonstrate an inverted tuning curve, meaning that, opposite to the pyramidal neurons, the activity of CB INs would decrease when a visual stimulus was presented. It was proposed that these CB INs were responsible for tuning the responses of pyramidal cells against distractors. These early proposals paved the way for more recent research utilizing modern cell-type specific manipulation techniques to discern the contribution of individual IN populations to working memory functions.

**Figure 2:**
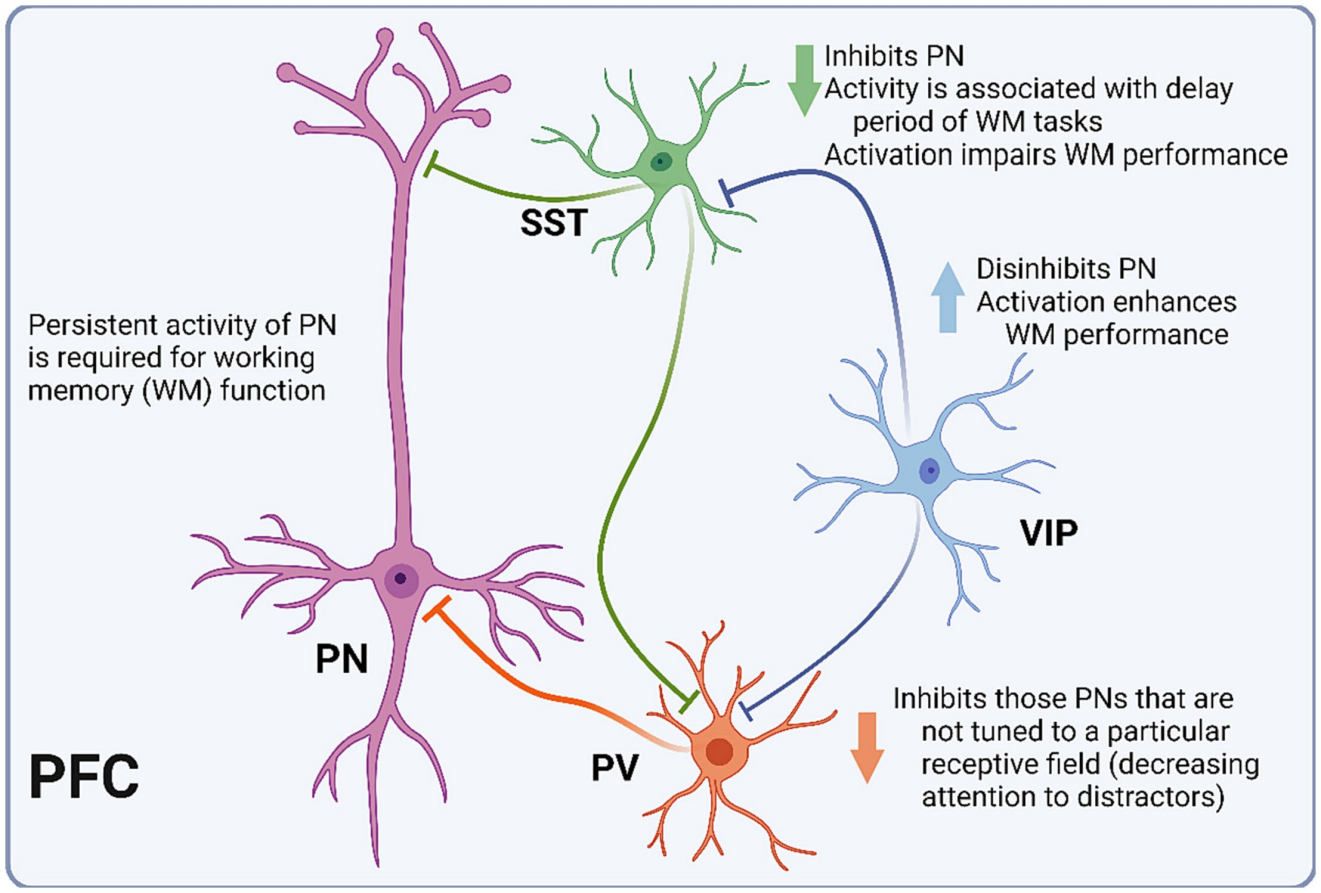
Schematic representation of PFC microcircuitry. Diagram depicting the microcircuitry of the pyramidal neuron (PN) with each of the three interneuron (IN) subtypes. Parvalbumin-expressing INs (PV) inhibit PNs that are not tuned to the relevant receptive field. Somatostatin-expressing INs (SST) inhibit PNs, and their activity is associated with the delay period of working memory (WM) tasks. Vasoactive intestinal peptide-expressing INs (VIP) inhibit PV and SST INs, thus disinhibiting PNs.

Today, PFC INs are usually categorized according to three main groups: parvalbumin-expressing (PV), somatostatin-expressing (SST), and 5-HT receptor 3 (5-HT3R)-expressing (Kepecs and Fishell, 2014; Yang et al., 2021). The electrophysiological and morphological diversity of PFC INs is not well contained by these groups. However, some generalizations can be made regarding spiking pattern and synapse location. Generally, PV INs are fast-spiking and innervate perisomatic areas of pyramidal neurons (Kvitsiani et al., 2013). SST INs are largely dendritic-targeting and demonstrate a wide range of electrophysiological properties, including strong facilitation of excitatory inputs (DeFelipe et al., 2013; Kepecs and Fishell, 2014). Lastly, 5-HT3R INs include INs expressing the vasoactive intestinal peptide (VIP) and neurogliaform cells (NGFCs) (Tremblay et al., 2016) (Figure 2). Whereas VIP INs have been shown to preferentially target other INs, resulting in pyramidal cell disinhibition, NGFCs are mostly confined to layer I, where they potently inhibit both local INs and pyramidal cells. In a 2015 study of PV-Cre mice, selective silencing of PV INs via expression of tetanus toxin light chain (TeLC) led to impairment in a hole-board test of working memory (Murray et al., 2015). In contrast, when TeLC was expressed in SST INs, mice did not demonstrate working memory impairment. Notably, TeLC-mediated inhibition results in a permanent and relatively complete reduction in activity of the target cell population, which may limit the interpretation of experimental data, as compensatory mechanisms that arise in response to such perturbations are difficult to account for.

Extracellular electrophysiological recordings cannot distinguish between IN populations with the precision afforded by Cre lines. However, cells can be roughly grouped into fast-spiking, regular-spiking, and irregular-spiking populations, corresponding to PV, SST, and VIP INs, respectively (DeFelipe et al., 2013; Kepecs and Fishell, 2014). In a 2016 study utilizing tetrode recordings of PFC neurons in mice undergoing a T-maze task of working memory, putative INs were categorized as putative PV or SST cells, and PV IN activity showed little coordination with the delay period compared to SST INs (Kim et al., 2016). Additionally, SST IN activity served as a better predictor of successful performance in the task, which supports the hypothesized role of SST INs in tuning the PFC functional column to maintain the persistent activity necessary for optimal performance. When optogenetic techniques were applied, SST activation impaired task performance while PV IN activation suppressed the activity of recorded pyramidal neurons, but did not significantly diminish task success. In a 2018 study, optogenetic inhibition of SST INs again resulted in working memory impairment, seemingly by decreasing PFC synchrony with the hippocampus (Abbas et al., 2018). In a Go-No-Go task of working memory in mice, Kamigaki and Dan (2017) demonstrated that inhibition of pyramidal neurons via PV IN activation resulted in significant decreases in task performance, while disinhibition of pyramidal neurons via activation of VIP INs enhanced behavioral performance.

While the precise role of the prefrontal microcircuit in working memory function is an area of active investigation, it is clear that different populations of INs have distinct and interconnected roles in the maintenance of persistent activity and subsequent task performance, as predicted by the Goldman-Rakic modeling from more than 20 years ago. PV INs potently inhibit pyramidal neurons, likely playing a role in downregulating the activity of those pyramidal neurons that are not tuned to a particular receptive field (Kim et al., 2016). SST INs improve task performance by inhibiting the conduction in distal dendrites of active pyramidal cells (Abbas et al., 2018). Finally, VIP INs potently disinhibit pyramidal neurons, maintaining the excitation level necessary for persistent activity (Kamigaki and Dan, 2017). Furthermore, interneuron dysfunction has been identified in a number of psychiatric diseases that also demonstrate working memory impairment, including Alzheimer’s disease (Palop and Mucke, 2016), schizophrenia (Dienel and Lewis, 2019), and autism (Nomura, 2021), amongst others.

## The intersection of working memory and social function

While much of Goldman-Rakic’s work sought to reveal the individual neuronal properties and circuit-level dynamics that underlie working memory, she also worked to uncover the functional consequences of working memory deficits in psychiatric disease (Arnsten, 2013). Working memory underlies many facets of daily functioning, including social functioning. As a prerequisite for normal social functioning, organisms must be able to consider multiple social cues within their environment, assess and remember the social status of others, and adapt to the continuous and changing demands of social interaction, all of which are facets of social cognition that require adequate working memory. Investigations of young children have revealed a relationship between the development of working memory capacity and social-relational functioning (de Wilde et al., 2016). A subset of working memory that deals specifically with the processing and integration of social information, known as social working memory, permits individuals to engage in “mentalizing,” or the consideration of other individuals’ thoughts, traits, and beliefs [(Meyer et al., 2012), reviewed in Meyer and Lieberman (2012)]. Deficits in working memory can prevent the consideration and manipulation of social information necessary for successful social behaviors, resulting in social deficits. Co-occurring deficits in working memory and social functioning are noted in a number of psychiatric and neurological conditions, including traumatic brain injury (Nolan et al., 2018), autism spectrum disorder (Rabiee et al., 2020; Gong et al., 2023; Memisevic et al., 2023), borderline personality disorder (Krause-Utz et al., 2014), epilepsy (Lim et al., 2007; Hernan et al., 2014), post-traumatic stress disorder (Sippel et al., 2021), neurodegenerative conditions (Legaz et al., 2023), and intellectual disability (Ducic et al., 2018). Here, we highlight the relationship between working memory deficits and social dysfunction in three psychiatric conditions: attention deficit hyperactivity disorder (ADHD), schizophrenia, and social anxiety, with a special emphasis on how PFC dysfunction may mediate this relationship.

ADHD is a psychiatric disorder characterized by persistent and maladaptive inattention and/or hyperactivity and impulsivity that most commonly presents in children but can exist across the lifespan (American Psychiatric Association, 2013). Studies investigating working memory in individuals with ADHD consistently but not ubiquitously (Schecklmann et al., 2010) find reduced working memory capacity in patients with ADHD compared to healthy controls (Ramos et al., 2020; Torgalsbøen et al., 2021; Friedman et al., 2023). Dysfunction and aberrant connectivity of the PFC have been implicated in the pathophysiology of ADHD, with functional imaging studies demonstrating decreased activation of the middle and right PFC during tasks of executive function (Yasumura et al., 2019) and decreased hemodynamic response in the dorsolateral PFC during working memory tasks (Friedman et al., 2023) in children with ADHD compared to neurotypical controls. The role of the PFC in ADHD symptomatology is further supported by the observed effects of current pharmacological treatments on PFC function and connectivity, and by the success of nonpharmacological intervention strategies that target the PFC. For example, functional magnetic resonance imaging has demonstrated a decrease in functional connectivity between the PFC and various subcortical brain regions during a working memory task in adolescents with ADHD who are currently taking stimulant medication compared to those same subjects without stimulant medication (Sheridan et al., 2010). Furthermore, a recent randomized, double-blind, sham-controlled trial of transcranial direct current stimulation (tDCS) in patients with ADHD demonstrated that tDCS directed over the PFC resulted in improved working memory in ADHD (Barham et al., 2022).

In addition to the established executive dysfunction and working memory deficits seen in ADHD, patients also demonstrate significant deficits in social functioning [(Caillies et al., 2014; Humphreys et al., 2016), reviewed by Nijmeijer et al. (2008)]. As ADHD is viewed primarily as a disorder of cognitive dysfunction, the resulting social deficits can be conceptualized as a consequence of working memory dysfunction, given appropriate testing paradigms. For example, Hilton and colleagues utilized a dual-task paradigm to investigate the relationship between working memory and social cue encoding in children with ADHD, and found that social cue encoding was significantly disrupted when working memory load was increased (Hilton et al., 2020). Furthermore, Kofler and colleagues found that working memory deficits may indirectly lead to social dysfunction in children with ADHD by impacting patients’ ability to focus on multiple environmental stimuli at one time to process and appropriately integrate social cues (Kofler et al., 2011). In a recent study examining the role of specific facets of executive dysfunction in social deficits of ADHD, Bullard and colleagues found that working memory mediated the relationship between social functioning and diagnosis (ADHD vs. typical development) based on teacher ratings (Bullard et al., 2024). An investigation of biological motion (BM) in children with ADHD found that deficits in both working memory and theory of mind, a necessary component of successful social functioning, were correlated with worse performance on the BM task, suggesting that an interplay between working memory and theory of mind dysfunction may be responsible for the deficits in social perception observed in ADHD (Imanipour et al., 2021). Animal models of ADHD have also demonstrated social deficits, with a neonatal homocysteine treatment model demonstrating increased hyperactivity, decreased sociability, and significant morphological changes in dendritic spine shape in numerous brain regions, including the PFC (De la Torre-Iturbe et al., 2022).

Perhaps one of the most debilitating psychiatric disorders, schizophrenia is characterized by positive symptoms (i.e., hallucinations, delusions), negative symptoms (e.g., anhedonia, avolition), and cognitive symptoms (e.g., executive dysfunction, working memory deficits) (American Psychiatric Association, 2013). Molecular, cellular, and circuit-level connectivity changes have been described across numerous brain regions in schizophrenia. Still, PFC dysfunction is arguably one of the most consistent findings in the study of schizophrenia pathophysiology (Gao et al., 2022). In examining schizophrenia, working memory deficits in particular, the PFC is extensively implicated. Functional imaging studies have demonstrated decreased PFC activation and reduced prefrontal-parietal communication during working memory tasks when compared to healthy controls (Deserno et al., 2012; Kumar et al., 2021). Furthermore, several imaging studies have supported the role of compensatory post-task PFC hyperactivation in the pathophysiology of working memory deficits in schizophrenia (Noda et al., 2017; Kumar et al., 2021). In a recent trial of tDCS in the treatment of schizophrenia, Meiron and colleagues found that tDCS targeted to the left PFC produced significant improvements in working memory in addition to a general reduction in symptom severity (Meiron et al., 2021). Mirroring results seen in the study of PFC-targeted tDCS treatment in patients with ADHD, the success of this intervention supports the role of PFC dysfunction in the pathophysiology of working memory deficits in schizophrenia.

As in ADHD, working memory deficits in schizophrenia may contribute to the observed social deficits of the disorder, and numerous studies have sought to explore the potential relationship between these symptom domains. Takahashi and colleagues found that spatial working memory dysfunction was correlated to various aspects of social dysfunction in schizophrenia, including community skills (Takahashi et al., 2005). Similarly, Huang and colleagues found that working memory dysfunction was able to predict corresponding dysfunction in social problem solving in a schizophrenic population (Huang et al., 2014). PFC dysfunction appears to mediate the relationship between working memory and social deficits in schizophrenia. Pu and colleagues found decreased lateral PFC activation compared to healthy controls during a working memory task, and found that activation in this region was significantly correlated with theory of mind scores in patients with schizophrenia (Pu et al., 2016).

Animal model studies have also supported a relationship between working memory deficits and social dysfunction in schizophrenia. Ibotenic acid-induced lesions of the CA1 region of the hippocampus result in cytoarchitectural changes in the PFC and amygdala of rodents, and this paradigm has been used to model schizophrenia symptoms, including working memory and social deficits (Martínez-Torres et al., 2021). In another rodent model of schizophrenia symptomatology, prenatal infection (poly I: C) results in impairments in working memory and reductions in social interaction, both of which are attenuated by treatment with cannabidiol (CBD) (Osborne et al., 2017). This is particularly interesting given the opposing effects of CBD and delta-9-tetrahydrocannabinol (THC), the two active ingredients in marijuana, on working memory and sociability when examined independently. In rats, intra-PFC infusion of THC resulted in increased anxiety-like behavior but no changes to working memory. In contrast, intra-PFC infusion of CBD resulted in impairments in working memory without corresponding changes in anxiety or sociability (Szkudlarek et al., 2019). Interestingly, however, intra-PFC infusion of CBD in the setting of acute PFC glutamatergic antagonism with MK-801 resulted in a reversal of the NMDAR antagonist-induced cognitive effects, suggesting that CBD may result in pro-cognitive and pro-social effects only in the setting of existing pathology, as was the case in the poly I: C model.

While both ADHD and schizophrenia serve as examples of psychiatric disorders in which PFC dysfunction and resulting working memory deficits may contribute to the observed social dysfunction, a reverse relationship, in which social stress or anxiety may lead to working memory deficits, has also been demonstrated. Acute social exclusion in healthy adolescent and young adult female participants results in decreased performance on working memory tasks (Xu et al., 2018; Fuhrmann et al., 2019). Additionally, adolescent social defeat stress has been shown to result in reductions in working memory performance in adulthood in rodent models (Novick et al., 2013; Weber et al., 2018). Furthermore, working memory appears to be altered in individuals with social anxiety, such that negative or socially threatening stimuli are preferentially retained at the expense of other social information, potentially resulting in decreased social functioning in these individuals (MacNamara et al., 2019; Yeung and Fernandes, 2019). Working memory may be specifically impaired in social anxiety under high-demand conditions, such as in tasks that require filtering and inhibition of irrelevant distractors (Moriya and Sugiura, 2012). Interestingly, results from preliminary studies suggest that working memory training may have therapeutic potential in individuals with social anxiety, further cementing the role of working memory in this disorder (Zhao et al., 2020).

## Conclusion

Working memory has a rich history within the fields of psychology, psychiatry, and neuroscience, and efforts to understand the neural mechanisms underlying working memory processes have additionally served to elucidate the structure and function of critical brain regions of higher cognitive function, most notably the PFC. From her early work utilizing anterograde and retrograde tracers to investigate PFC connectivity across cortical and subcortical regions to her later work examining the role of dopamine in the pathophysiology of schizophrenia, Patricia Goldman-Rakic was instrumental in furthering our understanding of working memory and its role in numerous functional domains. As the technology available to researchers has continued to expand, the neuroscientific community has built upon the work of Dr. Goldman-Rakic, and many of her early hypotheses continue to shape the direction of working memory research across the globe. As we continue to examine the role of working memory in human cognitive function, particularly the role of working memory dysfunction in the pathophysiology of various psychiatric conditions, we see the myriad ways this process is critical to cognition and behavior.

## Author contributions

LTF: Conceptualization, Supervision, Writing – review & editing, Data curation, Formal analysis, Writing – original draft. NB: Conceptualization, Writing – original draft, Writing – review & editing. VM: Writing – original draft, Writing – review & editing. JC: Writing – original draft, Writing – review & editing. W-JG: Writing – review & editing, Conceptualization, Funding acquisition, Project administration, Supervision.

## References

[ref1] Abbas A. I. Sundiang M. J. M. Henoch B. Morton M. P. Bolkan S. S. Park A. J.. (2018). Somatostatin interneurons facilitate hippocampal-prefrontal synchrony and prefrontal spatial encoding. Neuron 100, 926–939.e3. doi: 10.1016/j.neuron.2018.09.029, PMID: 30318409 PMC6262834

[ref2] American Psychiatric Association (2013) Diagnostic and statistical manual of mental disorders: DSM-5: American psychiatric association Washington, DC.

[ref3] Arnsten A. F. (2013). The neurobiology of thought: the groundbreaking discoveries of Patricia Goldman-Rakic 1937-2003. Cereb. Cortex 23, 2269–2281. doi: 10.1093/cercor/bht195 23926115 PMC3767966

[ref4] Arnsten A. F. T. (2023). Retrospective: Patricia S. Goldman-Rakic, pioneer in neuroscience and co-founder of the journal, cerebral cortex. Cereb. Cortex 33, 8089–8100. doi: 10.1093/cercor/bhad159, PMID: 37143181 PMC10321099

[ref5] Bannerman D. M. Rawlins J. N. McHugh S. B. Deacon R. M. Yee B. K. Bast T.. (2004). Regional dissociations within the hippocampus--memory and anxiety. Neurosci. Biobehav. Rev. 28, 273–283. doi: 10.1016/j.neubiorev.2004.03.004, PMID: 15225971

[ref6] Barak O. Tsodyks M. (2014). Working models of working memory. Curr. Opin. Neurobiol. 25, 20–24. doi: 10.1016/j.conb.2013.10.008 24709596

[ref7] Barham H. Büyükgök D. Aksu S. Soyata A. Z. Bulut G. Eskicioğlu G.. (2022). Evidence for modulation of planning and working memory capacities by transcranial direct current stimulation in a sample of adults with attention deficit hyperactivity disorder. Neurosci. Lett. 790:136883. doi: 10.1016/j.neulet.2022.136883, PMID: 36152744

[ref8] Bolkan S. S. Stujenske J. M. Parnaudeau S. Spellman T. J. Rauffenbart C. Abbas A. I.. (2017). Thalamic projections sustain prefrontal activity during working memory maintenance. Nat. Neurosci. 20, 987–996. doi: 10.1038/nn.4568, PMID: 28481349 PMC5501395

[ref9] Bullard C. C. Alderson R. M. Roberts D. K. Tatsuki M. O. Sullivan M. A. Kofler M. J. (2024). Social functioning in children with ADHD: an examination of inhibition, self-control, and working memory as potential mediators. Child Neuropsychol., 1–23. doi: 10.1080/09297049.2024.2304375, PMID: 38269494 PMC11269528

[ref10] Caillies S. Bertot V. Motte J. Raynaud C. Abely M. (2014). Social cognition in ADHD: irony understanding and recursive theory of mind. Res. Dev. Disabil. 35, 3191–3198. doi: 10.1016/j.ridd.2014.08.002, PMID: 25155741

[ref11] Cenquizca L. A. Swanson L. W. (2007). Spatial organization of direct hippocampal field CA1 axonal projections to the rest of the cerebral cortex. Brain Res. Rev. 56, 1–26. doi: 10.1016/j.brainresrev.2007.05.002, PMID: 17559940 PMC2171036

[ref12] Cholvin T. Loureiro M. Cassel R. Cosquer B. Geiger K. De Sa N. D.. (2013). The ventral midline thalamus contributes to strategy shifting in a memory task requiring both prefrontal cortical and hippocampal functions. J. Neurosci. 33, 8772–8783. doi: 10.1523/JNEUROSCI.0771-13.2013, PMID: 23678120 PMC6618831

[ref13] Christophel T. B. Klink P. C. Spitzer B. Roelfsema P. R. Haynes J. D. (2017). The distributed nature of working memory. Trends Cogn. Sci. 21, 111–124. doi: 10.1016/j.tics.2016.12.007 28063661

[ref14] Constantinidis C. Funahashi S. Lee D. Murray J. D. Qi X. L. Wang M.. (2018). Persistent spiking activity underlies working memory. J. Neurosci. 38, 7020–7028. doi: 10.1523/JNEUROSCI.2486-17.2018, PMID: 30089641 PMC6083457

[ref15] Constantinidis C. Williams G. V. Goldman-Rakic P. S. (2002). A role for inhibition in shaping the temporal flow of information in prefrontal cortex. Nat. Neurosci. 5, 175–180. doi: 10.1038/nn799, PMID: 11802172

[ref16] Dalton G. L. Ma L. M. Phillips A. G. Floresco S. B. (2011). Blockade of NMDA GluN2B receptors selectively impairs behavioral flexibility but not initial discrimination learning. Psychopharmacology 216, 525–535. doi: 10.1007/s00213-011-2246-z, PMID: 21384103

[ref17] De la Torre-Iturbe S. Vázquez-Roque R. A. De la Cruz-López F. Flores G. Garcés-Ramírez L. (2022). Dendritic and behavioral changes in rats neonatally treated with homocysteine; a proposal as an animal model to study the attention deficit hyperactivity disorder. J. Chem. Neuroanat. 119:102057. doi: 10.1016/j.jchemneu.2021.102057, PMID: 34871732

[ref18] de Wilde A. Koot H. M. van Lier P. A. (2016). Developmental links between Children’s working memory and their social relations with teachers and peers in the early school years. J. Abnorm. Child Psychol. 44, 19–30. doi: 10.1007/s10802-015-0053-4, PMID: 26219261 PMC4715126

[ref19] DeFelipe J. López-Cruz P. L. Benavides-Piccione R. Bielza C. Larrañaga P. Anderson S.. (2013). New insights into the classification and nomenclature of cortical GABAergic interneurons. Nat. Rev. Neurosci. 14, 202–216. doi: 10.1038/nrn3444, PMID: 23385869 PMC3619199

[ref20] Deserno L. Sterzer P. Wüstenberg T. Heinz A. Schlagenhauf F. (2012). Reduced prefrontal-parietal effective connectivity and working memory deficits in schizophrenia. J. Neurosci. 32, 12–20. doi: 10.1523/JNEUROSCI.3405-11.2012, PMID: 22219266 PMC6621317

[ref21] D’Esposito M. Postle B. R. (2015). The cognitive neuroscience of working memory. Annu. Rev. Psychol. 66, 115–142. doi: 10.1146/annurev-psych-010814-015031, PMID: 25251486 PMC4374359

[ref22] Dienel S. J. Lewis D. A. (2019). Alterations in cortical interneurons and cognitive function in schizophrenia. Neurobiol. Dis. 131:104208. doi: 10.1016/j.nbd.2018.06.020, PMID: 29936230 PMC6309598

[ref23] Dolleman-van der Weel M. J. Griffin A. L. Ito H. T. Shapiro M. L. Witter M. P. Vertes R. P.. (2019). The nucleus reuniens of the thalamus sits at the nexus of a hippocampus and medial prefrontal cortex circuit enabling memory and behavior. Learn. Mem. 26, 191–205. doi: 10.1101/lm.048389.118, PMID: 31209114 PMC6581009

[ref24] Duan A. R. Varela C. Zhang Y. Shen Y. Xiong L. Wilson M. A.. (2015). Delta frequency optogenetic stimulation of the thalamic nucleus reuniens is sufficient to produce working memory deficits: relevance to schizophrenia. Biol. Psychiatry 77, 1098–1107. doi: 10.1016/j.biopsych.2015.01.020, PMID: 25891221 PMC4444380

[ref25] Ducic B. Gligorovic M. Kaljaca S. (2018). Relation between working memory and self-regulation capacities and the level of social skills acquisition in people with moderate intellectual disability. J. Appl. Res. Intellect. Disabil. 31, 296–307. doi: 10.1111/jar.12385, PMID: 28707351

[ref26] Durstewitz D. Seamans J. K. Sejnowski T. J. (2000). Neurocomputational models of working memory. Nat. Neurosci. 3, 1184–1191. doi: 10.1038/81460 11127836

[ref27] Duvarci S. Simpson E. H. Schneider G. Kandel E. R. Roeper J. Sigurdsson T. (2018). Impaired recruitment of dopamine neurons during working memory in mice with striatal D2 receptor overexpression. Nat. Commun. 9:2822. doi: 10.1038/s41467-018-05214-4, PMID: 30026489 PMC6053467

[ref28] Fanselow M. S. Dong H. W. (2010). Are the dorsal and ventral hippocampus functionally distinct structures? Neuron 65, 7–19. doi: 10.1016/j.neuron.2009.11.031, PMID: 20152109 PMC2822727

[ref29] Ferguson B. R. Gao W. J. (2018). Thalamic control of cognition and social behavior via regulation of gamma-aminobutyric Acidergic signaling and excitation/inhibition balance in the medial prefrontal cortex. Biol. Psychiatry 83, 657–669. doi: 10.1016/j.biopsych.2017.11.033, PMID: 29373121 PMC5862785

[ref30] Friedman L. M. Eckrich S. J. Rapport M. D. Bohil C. J. Calub C. (2023). Working and short-term memory in children with ADHD: an examination of prefrontal cortical functioning using functional near-infrared spectroscopy (fNIRS). Child Neuropsychol. 18, 1–24. doi: 10.1080/09297049.2023.2213463, PMID: 37199502

[ref31] Fuhrmann D. Casey C. S. Speekenbrink M. Blakemore S. J. (2019). Social exclusion affects working memory performance in young adolescent girls. Dev. Cogn. Neurosci. 40:100718. doi: 10.1016/j.dcn.2019.100718, PMID: 31733525 PMC6905155

[ref32] Funahashi S. Bruce C. J. Goldman-Rakic P. S. (1989). Mnemonic coding of visual space in the monkey’s dorsolateral prefrontal cortex. J. Neurophysiol. 61, 331–349. doi: 10.1152/jn.1989.61.2.331, PMID: 2918358

[ref33] Funahashi S. Bruce C. J. Goldman-Rakic P. S. (1991). Neuronal activity related to saccadic eye movements in the monkey’s dorsolateral prefrontal cortex. J. Neurophysiol. 65, 1464–1483. doi: 10.1152/jn.1991.65.6.1464, PMID: 1875255

[ref34] Gao W. J. Yang S. S. Mack N. R. Chamberlin L. A. (2022). Aberrant maturation and connectivity of prefrontal cortex in schizophrenia-contribution of NMDA receptor development and hypofunction. Mol. Psychiatry 27, 731–743. doi: 10.1038/s41380-021-01196-w, PMID: 34163013 PMC8695640

[ref35] Ge C. Chen Z. Sun F. Hou R. Fan H. Li Y.. (2023) Timing-dependent modulation of working memory by VTA dopamine release in medial prefrontal cortex. bioRxiv:2023.2009. 2001.555676.

[ref36] Giguere M. Goldman-Rakic P. S. (1988). Mediodorsal nucleus: areal, laminar, and tangential distribution of afferents and efferents in the frontal lobe of rhesus monkeys. J. Comp. Neurol. 277, 195–213. doi: 10.1002/cne.902770204, PMID: 2466057

[ref37] Goldman P. S. Rosvold H. E. (1970). Localization of function within the dorsolateral prefrontal cortex of the rhesus monkey. Exp. Neurol. 27, 291–304. doi: 10.1016/0014-4886(70)90222-0 4987453

[ref38] Goldman-Rakic P. S. (1995). Cellular basis of working memory. Neuron 14, 477–485. doi: 10.1016/0896-6273(95)90304-6 7695894

[ref39] Goldman-Rakic P. S. (1999). The “psychic” neuron of the cerebral cortex. Ann. N. Y. Acad. Sci. 868, 13–26. doi: 10.1111/j.1749-6632.1999.tb11270.x 10414278

[ref40] Goldman-Rakic P. S. Friedman H. R. (1991). “The circuitry of working memory revealed by anatomy and metabolic imaging” in Frontal lobe function and dysfunction. eds. Levin H. S. Eisenberg H. M. Benton A. L. (Oxford: Oxford University Press)

[ref41] Goldman-Rakic P. S. Porrino L. J. (1985). The primate mediodorsal (MD) nucleus and its projection to the frontal lobe. J. Comp. Neurol. 242, 535–560. doi: 10.1002/cne.902420406, PMID: 2418080

[ref42] Goldman-Rakic P. S. Selemon L. D. Schwartz M. L. (1984). Dual pathways connecting the dorsolateral prefrontal cortex with the hippocampal formation and parahippocampal cortex in the rhesus monkey. Neuroscience 12, 719–743. doi: 10.1016/0306-4522(84)90166-0, PMID: 6472617

[ref43] Gong L. Guo D. Gao Z. Wei K. (2023). Atypical development of social and nonsocial working memory capacity among preschoolers with autism spectrum disorders. Autism Res. 16, 327–339. doi: 10.1002/aur.2853, PMID: 36374256

[ref44] Gordleeva S. Y. Tsybina Y. A. Krivonosov M. I. Ivanchenko M. V. Zaikin A. A. Kazantsev V. B.. (2021). Modeling working memory in a spiking neuron network accompanied by astrocytes. Front. Cell. Neurosci. 15:631485. doi: 10.3389/fncel.2021.631485, PMID: 33867939 PMC8044545

[ref45] Griffin A. L. (2015). Role of the thalamic nucleus reuniens in mediating interactions between the hippocampus and medial prefrontal cortex during spatial working memory. Front. Syst. Neurosci. 9:29. doi: 10.3389/fnsys.2015.00029 25805977 PMC4354269

[ref46] Hallock H. L. Wang A. Griffin A. L. (2016). Ventral midline thalamus is critical for hippocampal-prefrontal synchrony and spatial working memory. J. Neurosci. 36, 8372–8389. doi: 10.1523/JNEUROSCI.0991-16.2016, PMID: 27511010 PMC4978800

[ref47] Hallock H. L. Wang A. Shaw C. L. Griffin A. L. (2013). Transient inactivation of the thalamic nucleus reuniens and rhomboid nucleus produces deficits of a working-memory dependent tactile-visual conditional discrimination task. Behav. Neurosci. 127, 860–866. doi: 10.1037/a0034653, PMID: 24341710 PMC4009727

[ref48] Hembrook J. R. Mair R. G. (2011). Lesions of reuniens and rhomboid thalamic nuclei impair radial maze win-shift performance. Hippocampus 21, 815–826. doi: 10.1002/hipo.20797, PMID: 20572196 PMC2974946

[ref49] Hembrook J. R. Onos K. D. Mair R. G. (2012). Inactivation of ventral midline thalamus produces selective spatial delayed conditional discrimination impairment in the rat. Hippocampus 22, 853–860. doi: 10.1002/hipo.20945, PMID: 21542055

[ref50] Hernan A. E. Alexander A. Jenks K. R. Barry J. Lenck-Santini P. P. Isaeva E.. (2014). Focal epileptiform activity in the prefrontal cortex is associated with long-term attention and sociability deficits. Neurobiol. Dis. 63, 25–34. doi: 10.1016/j.nbd.2013.11.012, PMID: 24269731 PMC4397918

[ref51] Hilton D. C. Jarrett M. A. Rondon A. T. Tutek J. Mulla M. M. (2020). Increased working memory load in a dual-task design impairs nonverbal social encoding in children with high and low attention-deficit/hyperactivity disorder symptoms. Child Psychiatry Hum. Dev. 51, 127–137. doi: 10.1007/s10578-019-00915-3, PMID: 31359331

[ref52] Hoover W. B. Vertes R. P. (2012). Collateral projections from nucleus reuniens of thalamus to hippocampus and medial prefrontal cortex in the rat: a single and double retrograde fluorescent labeling study. Brain Struct. Funct. 217, 191–209. doi: 10.1007/s00429-011-0345-6, PMID: 21918815

[ref53] Huang J. Tan S. P. Walsh S. C. Spriggens L. K. Neumann D. L. Shum D. H.. (2014). Working memory dysfunctions predict social problem solving skills in schizophrenia. Psychiatry Res. 220, 96–101. doi: 10.1016/j.psychres.2014.07.043, PMID: 25110314

[ref54] Humphreys K. L. Galan C. A. Tottenham N. Lee S. S. (2016). Impaired social decision-making mediates the association between ADHD and social problems. J. Abnorm. Child Psychol. 44, 1023–1032. doi: 10.1007/s10802-015-0095-7, PMID: 26486935 PMC6613588

[ref55] Hyman J. M. Zilli E. A. Paley A. M. Hasselmo M. E. (2010). Working memory performance correlates with prefrontal-hippocampal Theta interactions but not with prefrontal neuron firing rates. Front. Integr. Neurosci. 4:2. doi: 10.3389/neuro.07.002.2010 20431726 PMC2861479

[ref56] Imanipour S. Sheikh M. Shayestefar M. Baloochnejad T. (2021). Deficits in working memory and theory of mind may underlie difficulties in social perception of children with ADHD. Neurol. Res. Int. 2021, 1–7. doi: 10.1155/2021/3793750 PMC8421162 34497727

[ref57] Isseroff A. Rosvold H. E. Galkin T. W. Goldman-Rakic P. S. (1982). Spatial memory impairments following damage to the mediodorsal nucleus of the thalamus in rhesus monkeys. Brain Res. 232, 97–113. doi: 10.1016/0006-8993(82)90613-8 7034865

[ref58] Jacob S. N. Nieder A. (2014). Complementary roles for primate frontal and parietal cortex in guarding working memory from distractor stimuli. Neuron 83, 226–237. doi: 10.1016/j.neuron.2014.05.009, PMID: 24991963

[ref59] Jones M. W. Wilson M. A. (2005). Theta rhythms coordinate hippocampal-prefrontal interactions in a spatial memory task. PLoS Biol. 3:e402. doi: 10.1371/journal.pbio.0030402, PMID: 16279838 PMC1283536

[ref60] Kamigaki T. Dan Y. (2017). Delay activity of specific prefrontal interneuron subtypes modulates memory-guided behavior. Nat. Neurosci. 20, 854–863. doi: 10.1038/nn.4554, PMID: 28436982 PMC5554301

[ref61] Kantrowitz J. T. Javitt D. C. (2010). N-methyl-d-aspartate (NMDA) receptor dysfunction or dysregulation: the final common pathway on the road to schizophrenia? Brain Res. Bull. 83, 108–121. doi: 10.1016/j.brainresbull.2010.04.006, PMID: 20417696 PMC2941541

[ref62] Kepecs A. Fishell G. (2014). Interneuron cell types are fit to function. Nature 505, 318–326. doi: 10.1038/nature12983, PMID: 24429630 PMC4349583

[ref63] Kievit J. Kuypers H. G. (1977). Organization of the thalamo-cortical connexions to the frontal lobe in the rhesus monkey. Exp. Brain Res. 29, 299–322. doi: 10.1007/BF00236173, PMID: 410652

[ref64] Kilonzo K. van der Veen B. Teutsch J. Schulz S. Kapanaiah S. K. T. Liss B.. (2021). Delayed-matching-to-position working memory in mice relies on NMDA-receptors in prefrontal pyramidal cells. Sci. Rep. 11:8788. doi: 10.1038/s41598-021-88200-z, PMID: 33888809 PMC8062680

[ref65] Kim D. Jeong H. Lee J. Ghim J. W. Her E. S. Lee S. H.. (2016). Distinct roles of Parvalbumin- and somatostatin-expressing interneurons in working memory. Neuron 92, 902–915. doi: 10.1016/j.neuron.2016.09.023, PMID: 27746132

[ref66] Kofler M. J. Rapport M. D. Bolden J. Sarver D. E. Raiker J. S. Alderson R. M. (2011). Working memory deficits and social problems in children with ADHD. J. Abnorm. Child Psychol. 39, 805–817. doi: 10.1007/s10802-011-9492-8 21468668

[ref67] Krause-Utz A. Elzinga B. M. Oei N. Y. Paret C. Niedtfeld I. Spinhoven P.. (2014). Amygdala and dorsal anterior cingulate connectivity during an emotional working memory task in borderline personality disorder patients with interpersonal trauma history. Front. Hum. Neurosci. 8:848. doi: 10.3389/fnhum.2014.00848 25389397 PMC4211399

[ref68] Kritzer M. F. Goldman-Rakic P. S. (1995). Intrinsic circuit organization of the major layers and sublayers of the dorsolateral prefrontal cortex in the rhesus monkey. J. Comp. Neurol. 359, 131–143. doi: 10.1002/cne.903590109 8557842

[ref69] Kumar V. Nichenmetla S. Chhabra H. Sreeraj V. S. Rao N. P. Kesavan M.. (2021). Prefrontal cortex activation during working memory task in schizophrenia: a fNIRS study. Asian J. Psychiatr. 56:102507. doi: 10.1016/j.ajp.2020.102507, PMID: 33388563

[ref9006] Kvitsiani D. Ranade S. Hangya B. Taniguchi H. Huang J. Z. Kepecs A. (2013). Distinct behavioural and network correlates of two interneuron types in prefrontal cortex. Nature 498, 363–366. 23708967 10.1038/nature12176 PMC4349584

[ref70] Laroche S. Davis S. Jay T. M. (2000). Plasticity at hippocampal to prefrontal cortex synapses: dual roles in working memory and consolidation. Hippocampus 10, 438–446. doi: 10.1002/1098-1063(2000)10:4<438::AID-HIPO10>3.0.CO;2-3, PMID: 10985283

[ref71] Layfield D. M. Patel M. Hallock H. Griffin A. L. (2015). Inactivation of the nucleus reuniens/rhomboid causes a delay-dependent impairment of spatial working memory. Neurobiol. Learn. Mem. 125, 163–167. doi: 10.1016/j.nlm.2015.09.007, PMID: 26391450 PMC4648689

[ref72] Legaz A. Prado P. Moguilner S. Baez S. Santamaria-Garcia H. Birba A.. (2023). Social and non-social working memory in neurodegeneration. Neurobiol. Dis. 183:106171. doi: 10.1016/j.nbd.2023.106171, PMID: 37257663 PMC11177282

[ref73] Liebe S. Hoerzer G. M. Logothetis N. K. Rainer G. (2012). Theta coupling between V4 and prefrontal cortex predicts visual short-term memory performance. Nat. Neurosci. 15, 456–462. doi: 10.1038/nn.3038 22286175

[ref74] Lim C. E. Turner L. H. Heinrichs S. C. (2007). Short-term social recognition memory deficit and atypical social and physiological stressor reactivity in seizure-susceptible El mice. Seizure 16, 59–68. doi: 10.1016/j.seizure.2006.10.006, PMID: 17116413

[ref75] MacNamara A. Jackson T. B. Fitzgerald J. M. Hajcak G. Phan K. L. (2019). Working memory load and negative picture processing: neural and behavioral associations with panic, social anxiety, and positive affect. Biol. Psychiatry Cogn. Neurosci. Neuroimaging. 4, 151–159. doi: 10.1016/j.bpsc.2018.04.005, PMID: 29805056 PMC6197936

[ref76] Maisson D. J. Gemzik Z. M. Griffin A. L. (2018). Optogenetic suppression of the nucleus reuniens selectively impairs encoding during spatial working memory. Neurobiol. Learn. Mem. 155, 78–85. doi: 10.1016/j.nlm.2018.06.010, PMID: 29940254

[ref77] Martínez-Torres N. I. Vázquez-Hernández N. Martín-Amaya-Barajas F. L. Flores-Soto M. González-Burgos I. (2021). Ibotenic acid induced lesions impair the modulation of dendritic spine plasticity in the prefrontal cortex and amygdala, a phenomenon that underlies working memory and social behavior. Eur. J. Pharmacol. 896:173883. doi: 10.1016/j.ejphar.2021.173883, PMID: 33513334

[ref78] Meiron O. David J. Yaniv A. (2021). Left prefrontal transcranial direct-current stimulation reduces symptom-severity and acutely enhances working memory in schizophrenia. Neurosci. Lett. 755:135912. doi: 10.1016/j.neulet.2021.135912, PMID: 33894334

[ref79] Memisevic H. Pasalic A. Saletovic A. (2023). Autism severity level affects working memory and planning but not inhibition, shifting and emotional control. Autism Res. 16, 1335–1343. doi: 10.1002/aur.2952, PMID: 37212537

[ref80] Meyer M. L. Lieberman M. D. (2012). Social working memory: neurocognitive networks and directions for future research. Front. Psychol. 3:571. doi: 10.3389/fpsyg.2012.00571 23267340 PMC3527735

[ref81] Meyer M. L. Spunt R. P. Berkman E. T. Taylor S. E. Lieberman M. D. (2012). Evidence for social working memory from a parametric functional MRI study. Proc. Natl. Acad. Sci. USA 109, 1883–1888. doi: 10.1073/pnas.1121077109, PMID: 22308468 PMC3277536

[ref82] Monaco S. A. Gulchina Y. Gao W. J. (2015). NR2B subunit in the prefrontal cortex: a double-edged sword for working memory function and psychiatric disorders. Neurosci. Biobehav. Rev. 56, 127–138. doi: 10.1016/j.neubiorev.2015.06.022, PMID: 26143512 PMC4567400

[ref83] Mongillo G. Barak O. Tsodyks M. (2008). Synaptic theory of working memory. Science 319, 1543–1546. doi: 10.1126/science.1150769 18339943

[ref84] Moriya J. Sugiura Y. (2012). High visual working memory capacity in trait social anxiety. PLoS One 7:e34244. doi: 10.1371/journal.pone.0034244, PMID: 22496783 PMC3322141

[ref85] Murphy B. C. O’Reilly R. L. Singh S. M. (2005). Site-specific cytosine methylation in S-COMT promoter in 31 brain regions with implications for studies involving schizophrenia. Am. J. Med. Genet. B Neuropsychiatr. Genet. 133B, 37–42. doi: 10.1002/ajmg.b.30134, PMID: 15635661

[ref86] Murray J. D. Jaramillo J. Wang X. J. (2017). Working memory and decision-making in a Frontoparietal circuit model. J. Neurosci. 37, 12167–12186. doi: 10.1523/JNEUROSCI.0343-17.2017, PMID: 29114071 PMC5729190

[ref87] Murray A. J. Woloszynowska-Fraser M. U. Ansel-Bollepalli L. Cole K. L. Foggetti A. Crouch B.. (2015). Parvalbumin-positive interneurons of the prefrontal cortex support working memory and cognitive flexibility. Sci. Rep. 5:16778. doi: 10.1038/srep16778, PMID: 26608841 PMC4660359

[ref88] Nijmeijer J. S. Minderaa R. B. Buitelaar J. K. Mulligan A. Hartman C. A. Hoekstra P. J. (2008). Attention-deficit/hyperactivity disorder and social dysfunctioning. Clin. Psychol. Rev. 28, 692–708. doi: 10.1016/j.cpr.2007.10.003 18036711

[ref89] Noda T. Nakagome K. Setoyama S. Matsushima E. (2017). Working memory and prefrontal/temporal hemodynamic responses during post-task period in patients with schizophrenia: a multi-channel near-infrared spectroscopy study. J. Psychiatr. Res. 95, 288–298. doi: 10.1016/j.jpsychires.2017.09.001, PMID: 28934615

[ref90] Nolan A. Hennessy E. Krukowski K. Guglielmetti C. Chaumeil M. M. Sohal V. S.. (2018). Repeated mild head injury leads to wide-ranging deficits in higher-order cognitive functions associated with the prefrontal cortex. J. Neurotrauma 35, 2425–2434. doi: 10.1089/neu.2018.5731, PMID: 29732949 PMC6196749

[ref91] Nomura T. (2021). Interneuron dysfunction and inhibitory deficits in autism and fragile X syndrome. Cells 10:2610. doi: 10.3390/cells10102610, PMID: 34685590 PMC8534049

[ref92] Novick A. M. Miiller L. C. Forster G. L. Watt M. J. (2013). Adolescent social defeat decreases spatial working memory performance in adulthood. Behav. Brain Funct. 9:39. doi: 10.1186/1744-9081-9-39, PMID: 24134918 PMC3853352

[ref93] O’Neill P. K. Gordon J. A. Sigurdsson T. (2013). Theta oscillations in the medial prefrontal cortex are modulated by spatial working memory and synchronize with the hippocampus through its ventral subregion. J. Neurosci. 33, 14211–14224. doi: 10.1523/JNEUROSCI.2378-13.2013, PMID: 23986255 PMC3756763

[ref94] Osborne A. L. Solowij N. Babic I. Huang X. F. Weston-Green K. (2017). Improved social interaction, recognition and working memory with Cannabidiol treatment in a prenatal infection (poly I:C) rat model. Neuropsychopharmacology 42, 1447–1457. doi: 10.1038/npp.2017.40, PMID: 28230072 PMC5436124

[ref95] Oyama K. Hori Y. Nagai Y. Miyakawa N. Mimura K. Hirabayashi T.. (2021). Chemogenetic dissection of the primate prefronto-subcortical pathways for working memory and decision-making. Sci. Adv. 7:eabg4246. doi: 10.1126/sciadv.abg4246, PMID: 34162548 PMC8221616

[ref96] Palop J. J. Mucke L. (2016). Network abnormalities and interneuron dysfunction in Alzheimer disease. Nat. Rev. Neurosci. 17, 777–792. doi: 10.1038/nrn.2016.141, PMID: 27829687 PMC8162106

[ref97] Parnaudeau S. Bolkan S. S. Kellendonk C. (2018). The Mediodorsal thalamus: an essential partner of the prefrontal cortex for cognition. Biol. Psychiatry 83, 648–656. doi: 10.1016/j.biopsych.2017.11.008, PMID: 29275841 PMC5862748

[ref98] Parnaudeau S. O’Neill P. K. Bolkan S. S. Ward R. D. Abbas A. I. Roth B. L.. (2013). Inhibition of mediodorsal thalamus disrupts thalamofrontal connectivity and cognition. Neuron 77, 1151–1162. doi: 10.1016/j.neuron.2013.01.038, PMID: 23522049 PMC3629822

[ref99] Pasternak T. Greenlee M. W. (2005). Working memory in primate sensory systems. Nat. Rev. Neurosci. 6, 97–107. doi: 10.1038/nrn1603 15654324

[ref100] Pu S. Nakagome K. Yamada T. Itakura M. Yamanashi T. Yamada S.. (2016). Social cognition and prefrontal hemodynamic responses during a working memory task in schizophrenia. Sci. Rep. 6:22500. doi: 10.1038/srep22500, PMID: 26928254 PMC4772542

[ref101] Rabiee A. Vasaghi-Gharamaleki B. Samadi S. A. Amiri-Shavaki Y. Alaghband-Rad J. (2020). Working memory deficits and its relationship to autism Spectrum disorders. Iran J Med Sci 45, 100–109. doi: 10.30476/IJMS.2019.45315, PMID: 32210486 PMC7071553

[ref102] Ramos A. A. Hamdan A. C. Machado L. (2020). A meta-analysis on verbal working memory in children and adolescents with ADHD. Clin. Neuropsychol. 34, 873–898. doi: 10.1080/13854046.2019.1604998, PMID: 31007130

[ref103] Riley M. R. Constantinidis C. (2015). Role of prefrontal persistent activity in working memory. Front. Syst. Neurosci. 9:181. doi: 10.3389/fnsys.2015.00181 26778980 PMC4700146

[ref104] Rudy B. Fishell G. Lee S. Hjerling-Leffler J. (2011). Three groups of interneurons account for nearly 100% of neocortical GABAergic neurons. Dev. Neurobiol. 71, 45–61. doi: 10.1002/dneu.20853, PMID: 21154909 PMC3556905

[ref105] Salazar R. F. Dotson N. M. Bressler S. L. Gray C. M. (2012). Content-specific fronto-parietal synchronization during visual working memory. Science 338, 1097–1100. doi: 10.1126/science.1224000, PMID: 23118014 PMC4038369

[ref106] Salimi M. Tabasi F. Nazari M. Ghazvineh S. Raoufy M. R. (2022). The olfactory bulb coordinates the ventral hippocampus-medial prefrontal cortex circuit during spatial working memory performance. J. Physiol. Sci. 72:9. doi: 10.1186/s12576-022-00833-5, PMID: 35468718 PMC10717655

[ref107] Sawaguchi T. Goldman-Rakic P. S. (1994). The role of D1-dopamine receptor in working memory: local injections of dopamine antagonists into the prefrontal cortex of rhesus monkeys performing an oculomotor delayed-response task. J. Neurophysiol. 71, 515–528. doi: 10.1152/jn.1994.71.2.515, PMID: 7909839

[ref108] Schecklmann M. Romanos M. Bretscher F. Plichta M. M. Warnke A. Fallgatter A. J. (2010). Prefrontal oxygenation during working memory in ADHD. J. Psychiatr. Res. 44, 621–628. doi: 10.1016/j.jpsychires.2009.11.018, PMID: 20044098

[ref109] Seamans J. K. Yang C. R. (2004). The principal features and mechanisms of dopamine modulation in the prefrontal cortex. Prog. Neurobiol. 74, 1–58. doi: 10.1016/j.pneurobio.2004.05.006, PMID: 15381316

[ref110] Sheridan M. A. Hinshaw S. D’Esposito M. (2010). Stimulant medication and prefrontal functional connectivity during working memory in ADHD: a preliminary report. J. Atten. Disord. 14, 69–78. doi: 10.1177/1087054709347444, PMID: 20576647 PMC2935299

[ref111] Sigurdsson T. Stark K. L. Karayiorgou M. Gogos J. A. Gordon J. A. (2010). Impaired hippocampal-prefrontal synchrony in a genetic mouse model of schizophrenia. Nature 464, 763–767. doi: 10.1038/nature08855, PMID: 20360742 PMC2864584

[ref112] Sippel L. M. Holtzheimer P. E. Huckins J. F. Collier E. Feilong M. Wheatley T.. (2021). Neurocognitive mechanisms of poor social connection in posttraumatic stress disorder: evidence for abnormalities in social working memory. Depress. Anxiety 38, 615–625. doi: 10.1002/da.23139, PMID: 33621379 PMC8169539

[ref113] Spellman T. Rigotti M. Ahmari S. E. Fusi S. Gogos J. A. Gordon J. A. (2015). Hippocampal-prefrontal input supports spatial encoding in working memory. Nature 522, 309–314. doi: 10.1038/nature14445, PMID: 26053122 PMC4505751

[ref114] Strange B. A. Witter M. P. Lein E. S. Moser E. I. (2014). Functional organization of the hippocampal longitudinal axis. Nat. Rev. Neurosci. 15, 655–669. doi: 10.1038/nrn3785 25234264

[ref115] Szkudlarek H. J. Desai S. J. Renard J. Pereira B. Norris C. Jobson C. E. L.. (2019). Delta-9-tetrahydrocannabinol and Cannabidiol produce dissociable effects on prefrontal cortical executive function and regulation of affective behaviors. Neuropsychopharmacology 44, 817–825. doi: 10.1038/s41386-018-0282-7, PMID: 30538288 PMC6372719

[ref116] Takahashi H. Iwase M. Nakahachi T. Sekiyama R. Tabushi K. Kajimoto O.. (2005). Spatial working memory deficit correlates with disorganization symptoms and social functioning in schizophrenia. Psychiatry Clin. Neurosci. 59, 453–460. doi: 10.1111/j.1440-1819.2005.01398.x, PMID: 16048451

[ref117] Thierry A. M. Gioanni Y. Degenetais E. Glowinski J. (2000). Hippocampo-prefrontal cortex pathway: anatomical and electrophysiological characteristics. Hippocampus 10, 411–419. doi: 10.1002/1098-1063(2000)10:4<411::AID-HIPO7>3.0.CO;2-A, PMID: 10985280

[ref118] Torgalsbøen B. R. Zeiner P. Øie M. G. (2021). Pre-attention and working memory in ADHD: a 25-year follow-up study. J. Atten. Disord. 25, 895–905. doi: 10.1177/1087054719879491, PMID: 31625421

[ref9007] Tremblay R. Lee S. Rudy B. (2016). GABAergic interneurons in the neocortex: from cellular properties to circuits. Neuron 91, 260–292. 27477017 10.1016/j.neuron.2016.06.033 PMC4980915

[ref119] Varela C. Kumar S. Yang J. Y. Wilson M. A. (2014). Anatomical substrates for direct interactions between hippocampus, medial prefrontal cortex, and the thalamic nucleus reuniens. Brain Struct. Funct. 219, 911–929. doi: 10.1007/s00429-013-0543-5, PMID: 23571778 PMC4179252

[ref120] Vertes R. P. (2002). Analysis of projections from the medial prefrontal cortex to the thalamus in the rat, with emphasis on nucleus reuniens. J. Comp. Neurol. 442, 163–187. doi: 10.1002/cne.10083 11754169

[ref121] Vertes R. P. (2006). Interactions among the medial prefrontal cortex, hippocampus and midline thalamus in emotional and cognitive processing in the rat. Neuroscience 142, 1–20. doi: 10.1016/j.neuroscience.2006.06.027, PMID: 16887277

[ref122] Vertes R. P. Hoover W. B. Do Valle A. C. Sherman A. Rodriguez J. J. (2006). Efferent projections of reuniens and rhomboid nuclei of the thalamus in the rat. J. Comp. Neurol. 499, 768–796. doi: 10.1002/cne.21135, PMID: 17048232

[ref123] Viena T. D. Linley S. B. Vertes R. P. (2018). Inactivation of nucleus reuniens impairs spatial working memory and behavioral flexibility in the rat. Hippocampus 28, 297–311. doi: 10.1002/hipo.22831, PMID: 29357198 PMC5871605

[ref124] Wager T. D. Smith E. E. (2003). Neuroimaging studies of working memory: a meta-analysis. Cogn. Affect. Behav. Neurosci. 3, 255–274. doi: 10.3758/CABN.3.4.255 15040547

[ref125] Wang X. J. (1999). Synaptic basis of cortical persistent activity: the importance of NMDA receptors to working memory. J. Neurosci. 19, 9587–9603. doi: 10.1523/JNEUROSCI.19-21-09587.1999, PMID: 10531461 PMC6782911

[ref126] Wang M. Gamo N. J. Yang Y. Jin L. E. Wang X. J. Laubach M.. (2011). Neuronal basis of age-related working memory decline. Nature 476, 210–213. doi: 10.1038/nature10243, PMID: 21796118 PMC3193794

[ref127] Wang M. Ramos B. P. Paspalas C. D. Shu Y. Simen A. Duque A.. (2007). Alpha2A-adrenoceptors strengthen working memory networks by inhibiting cAMP-HCN channel signaling in prefrontal cortex. Cell 129, 397–410. doi: 10.1016/j.cell.2007.03.015, PMID: 17448997

[ref128] Wang H. Stradtman G. G. Wang X. J. Gao W. J. (2008). A specialized NMDA receptor function in layer 5 recurrent microcircuitry of the adult rat prefrontal cortex. Proc. Natl. Acad. Sci. USA 105, 16791–16796. doi: 10.1073/pnas.0804318105, PMID: 18922773 PMC2575498

[ref129] Wang X. J. Tegner J. Constantinidis C. Goldman-Rakic P. S. (2004). Division of labor among distinct subtypes of inhibitory neurons in a cortical microcircuit of working memory. Proc. Natl. Acad. Sci. USA 101, 1368–1373. doi: 10.1073/pnas.0305337101, PMID: 14742867 PMC337059

[ref130] Wang M. Yang Y. Wang C. J. Gamo N. J. Jin L. E. Mazer J. A.. (2013). NMDA receptors subserve persistent neuronal firing during working memory in dorsolateral prefrontal cortex. Neuron 77, 736–749. doi: 10.1016/j.neuron.2012.12.032, PMID: 23439125 PMC3584418

[ref131] Weber M. A. Graack E. T. Scholl J. L. Renner K. J. Forster G. L. Watt M. J. (2018). Enhanced dopamine D2 autoreceptor function in the adult prefrontal cortex contributes to dopamine hypoactivity following adolescent social stress. Eur. J. Neurosci. 48, 1833–1850. doi: 10.1111/ejn.14019, PMID: 29904960 PMC6105450

[ref132] Wouterlood F. G. Saldana E. Witter M. P. (1990). Projection from the nucleus reuniens thalami to the hippocampal region: light and electron microscopic tracing study in the rat with the anterograde tracer *Phaseolus vulgaris*-leucoagglutinin. J. Comp. Neurol. 296, 179–203. doi: 10.1002/cne.902960202, PMID: 2358531

[ref133] Xu M. Qiao L. Qi S. Li Z. Diao L. Fan L.. (2018). Social exclusion weakens storage capacity and attentional filtering ability in visual working memory. Soc. Cogn. Affect. Neurosci. 13, 92–101. doi: 10.1093/scan/nsx139, PMID: 29149349 PMC5793715

[ref9008] Yang S. S. Mack N. R. Shu Y. Gao W. J. (2021). Prefrontal GABAergic interneurons gate long-range afferents to regulate prefrontal cortex-associated complex behaviors. Front Neural Circuits 15:716408. 34322002 10.3389/fncir.2021.716408 PMC8313241

[ref134] Yasumura A. Omori M. Fukuda A. Takahashi J. Yasumura Y. Nakagawa E.. (2019). Age-related differences in frontal lobe function in children with ADHD. Brain and Development 41, 577–586. doi: 10.1016/j.braindev.2019.03.006 30952459

[ref135] Yeung R. C. Fernandes M. A. (2019). Altered working memory capacity for social threat words in high versus low social anxiety. Anxiety Stress Coping 32, 505–521. doi: 10.1080/10615806.2019.1626838, PMID: 31232101

[ref136] Zhao X. Dang C. Maes J. H. R. (2020). Effects of working memory training on EEG, cognitive performance, and self-report indices potentially relevant for social anxiety. Biol. Psychol. 150:107840. doi: 10.1016/j.biopsycho.2019.107840, PMID: 31904404

